# Isolated cardiac sarcoidosis presenting as tumour-like multiple cardiac masses with massive pericardial effusion

**DOI:** 10.1093/ehjcr/ytag333

**Published:** 2026-05-12

**Authors:** Mayu Yamada, Katsumi Ueno

**Affiliations:** Department of Cardiology, Matsunami General Hospital, 185-1 Tashiro, Kasamatsu, Gifu 501-6062, Japan; Department of Cardiology, Matsunami General Hospital, 185-1 Tashiro, Kasamatsu, Gifu 501-6062, Japan

## Case description

**Figure 1:**
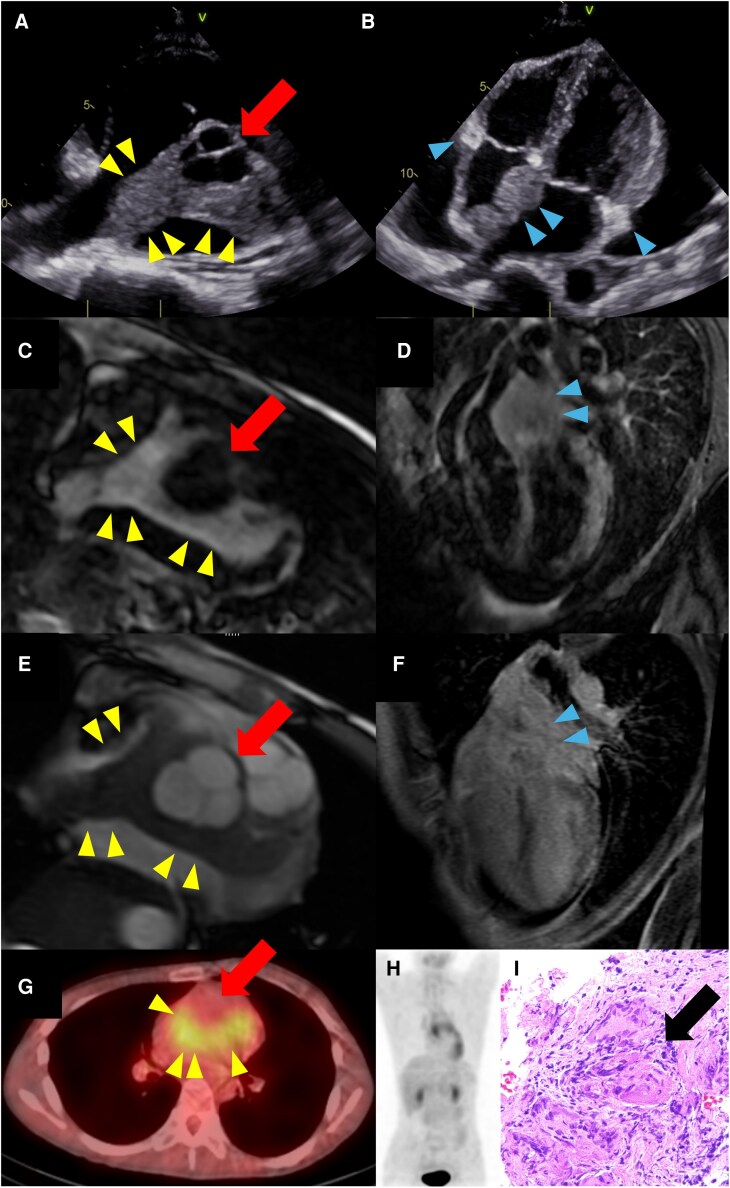
(*A*) Transthoracic echocardiography, parasternal short-axis view at the level of the aortic valve, demonstrating mass-like myocardial infiltration (yellow arrowheads) surrounding the aortic root (red arrow). (*B*) Transthoracic echocardiography, apical four-chamber view, showing multiple mass-like lesions involving the atrial septum (blue arrowheads) and the annuli of the mitral and tricuspid valves. (*C*) Cardiac magnetic resonance imaging (CMR), T2-weighted short tau inversion recovery (T2-STIR), short-axis view at the level of the aortic root, demonstrating high signal intensity in the myocardium surrounding the aortic root (yellow arrowheads), consistent with active myocardial inflammation. (*D*) CMR, T2-STIR, four-chamber view, showing high signal intensity in the atrial septum and periannular regions (blue arrowheads), consistent with the mass-like lesions identified on echocardiography. (*E*) CMR, late gadolinium enhancement (LGE), short-axis view at the level of the aortic root, demonstrating limited and heterogeneous enhancement within the mass-like infiltrative region (yellow arrowheads). (*F*) CMR, LGE, four-chamber view, showing heterogeneous enhancement in the interatrial septum and periannular regions (blue arrowheads). (*G*) 18F-fluorodeoxyglucose positron emission tomography (FDG-PET), axial view, demonstrating focal myocardial uptake consistent with the mass-like lesions identified on echocardiography and CMR (yellow arrowheads). (*H*) FDG-PET, whole-body image, demonstrating no abnormal extracardiac uptake, indicating that FDG accumulation was confined to the heart. (*I*) Endomyocardial biopsy specimen stained with haematoxylin and eosin, demonstrating non-caseating granulomas (black arrow) composed of epithelioid cells and multinucleated giant cells, consistent with sarcoidosis (original magnification ×400).

A 30-year-old man presented with a 2-month history of progressive dysphagia and palpitations. He had a blood pressure of 117/72 mmHg, pulse rate of 86 beats/min, and oxygen saturation of 96%. Electrocardiography showed a first-degree atrioventricular block (*PR* 366 ms). Computed tomography revealed massive pericardial and bilateral pleural effusions without lymphadenopathy or extracardiac masses. Transthoracic echocardiography demonstrated mass-like myocardial infiltration surrounding the aortic root, as well as multiple mass lesions involving the interatrial septum and the mitral and tricuspid annuli (*Figure 1A* and *B*, Supplementary material online, *Video S1*). The day after admission, he developed cardiac tamponade and underwent pericardial drainage. The pericardial fluid was light yellow, with negative cytology and cultures. Because malignancy was suspected, 18F-fluorodeoxyglucose positron emission tomography (FDG-PET) was performed to assess systemic involvement. FDG-PET revealed increased uptake confined to the heart, with no evidence of extracardiac disease (*Figure 1G and H*). Subsequently, cardiac magnetic resonance imaging demonstrated more extensive high signal intensity on T2-weighted black-blood short tau inversion recovery images, with limited and heterogeneous late gadolinium enhancement, suggesting predominantly active myocardial inflammation rather than fibrosis (*Figure 1C–F*). On Day 14, endomyocardial biopsy was performed under transoesophageal echocardiographic guidance (see Supplementary material online, *Video S2*). Recurrent tamponade occurred on Day 16, requiring repeat drainage; the pericardial fluid was not haemorrhagic. The PR interval progressively prolonged with haemodynamic instability. On Day 22, because of suspected malignant lymphoma and clinical deterioration, prednisolone of 100 mg was administered. Laboratory and histological findings revealed: angiotensin-converting enzyme 25.1 U/L (normal: 8.3–21.4), soluble IL-2 receptor 2170 U/L (normal: 157–474), and non-caseating granulomas (*Figure 1I*). He was diagnosed with isolated cardiac sarcoidosis (CS). One week later, echocardiography revealed resolution of cardiac lesions and pericardial effusion (see Supplementary material online, *Video S3*). No ventricular arrhythmias were documented, and left ventricular function was preserved; therefore, device implantation was deferred. The PR interval shortened to 200 ms (see Supplementary material online, *Figure S1*), and he was discharged on Day 69. Follow-up FDG-PET showed no evidence of recurrent myocardial uptake (see Supplementary material online, *Figure S2*).

This case highlights that isolated CS can mimic malignancy by presenting as tumour-like intracardiac masses with cardiac tamponade, requiring multimodality imaging and histological confirmation for accurate diagnosis.^1–3^

## Supplementary Material

ytag333_Supplementary_Data

## Data Availability

The data underlying this article are available in the article and its online Supplementary material. Further inquiries can be shared on reasonable request to the corresponding author.

## Notes

**Consent:** The authors confirm that written consent for the submission and publication of this case report, including image(s) and associated text, has been obtained from the patient in line with the COPE guidance.
