# Supplementary figures and images for: Isolated cardiac sarcoidosis presenting as tumour-like multiple cardiac masses with massive pericardial effusion
Source: Eur Heart J Case Rep. 2026 May 12;10(5):ytag333. doi: 10.1093/ehjcr/ytag333 (PMC13187643; doi:10.1093/ehjcr/ytag333)

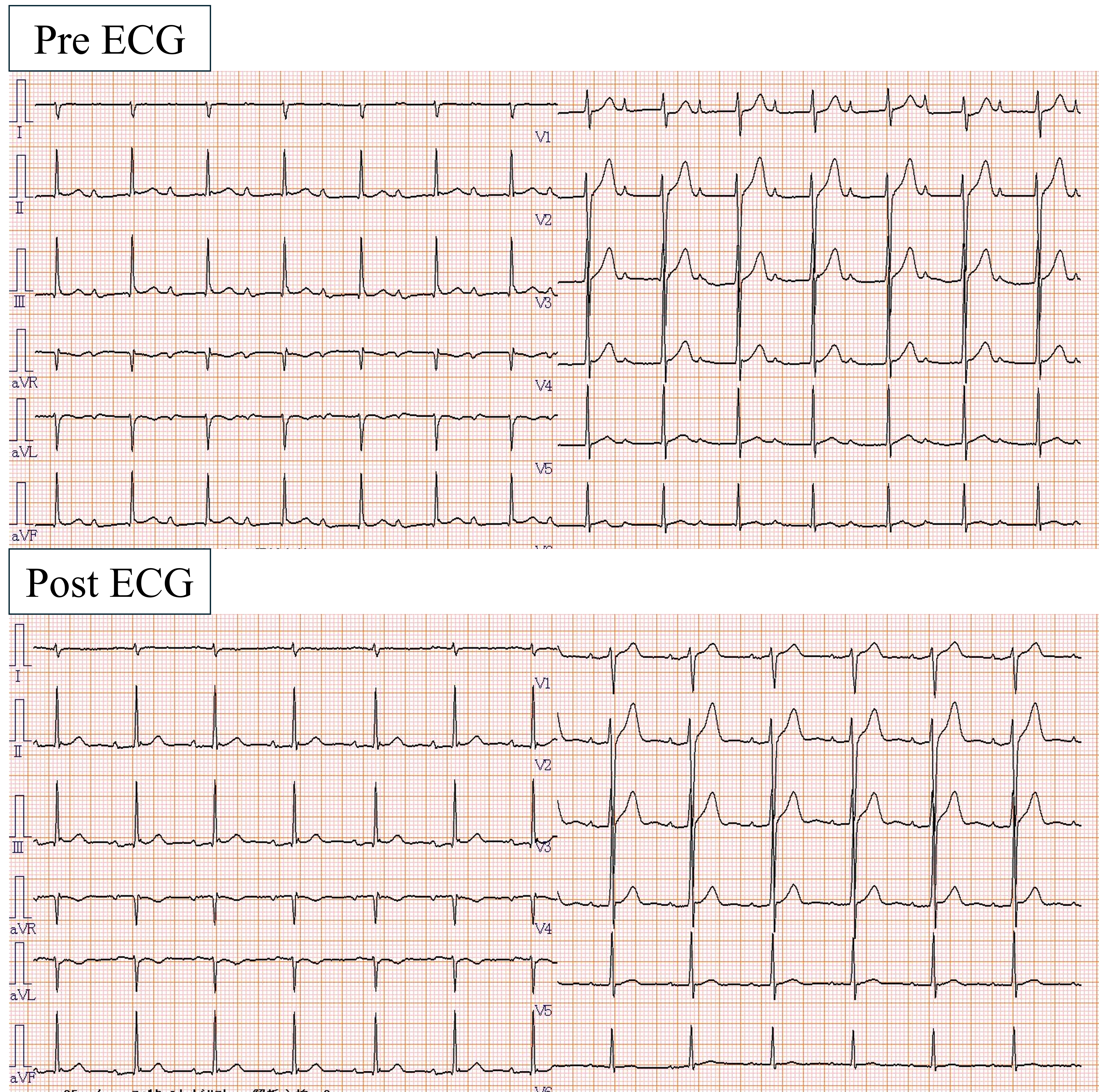

Supplement: ytag333_Supplementary_Data [file ytag333_supplementary_data.zip › 26-00373R1_renamed_b1c07.tif]

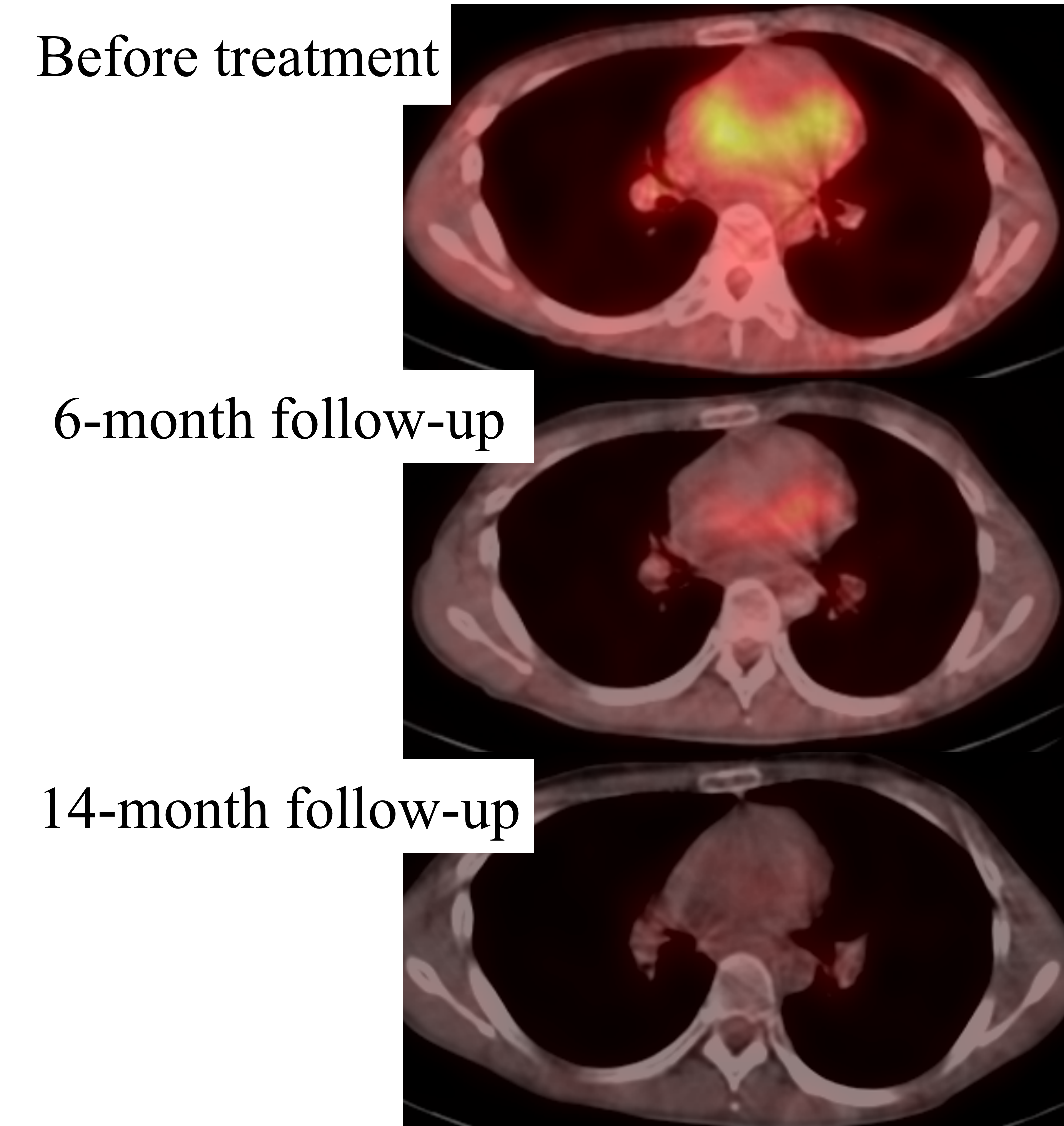

Supplement: ytag333_Supplementary_Data [file ytag333_supplementary_data.zip › Supplementary Figure2 PET follow-up.tif]
